# The BTLA and PD‐1 signaling pathways independently regulate the proliferation and cytotoxicity of human peripheral blood γδ T cells

**DOI:** 10.1002/iid3.390

**Published:** 2020-12-17

**Authors:** Hyun J. Hwang, Jae J. Lee, Sung H. Kang, Jin K. Suh, Eun S. Choi, Seongsoo Jang, Sang‐Hyun Hwang, Kyung‐Nam Koh, Ho J. Im, Nayoung Kim

**Affiliations:** ^1^ Asan Institute for Life Sciences and Department of Convergence Medicine, Asan Medical Center University of Ulsan College of Medicine Seoul Republic of Korea; ^2^ Asan Medical Institute for Convergence Science and Technology Asan Medical Center Seoul Republic of Korea; ^3^ Department of Pediatrics, Asan Medical Center University of Ulsan College of Medicine Seoul Republic of Korea; ^4^ Department of Laboratory Medicine, Asan Medical Center University of Ulsan College of Medicine Seoul Republic of Korea

**Keywords:** AKT, BTLA, cytotoxicity, human peripheral blood γδ T cells, PD‐1, proliferation, SHP2

## Abstract

**Background:**

B‐ and T‐lymphocyte attenuator (BTLA) and programmed cell death‐1 (PD‐1) inhibit γδ T cell homeostasis and activation. This study aimed to determine whether BTLA and PD‐1 signaling pathways were convergent or independent in human peripheral blood γδ T cells. Herein we demonstrate that the signalings of BTLA and PD‐1 regulated proliferation and cytotoxicity of human γδ T cells, respectively.

**Methods:**

Human peripheral blood γδ T cells were cultured with inactivated Jurkat cells in the presence of interleukin‐2 and zoledronate (Zol) for 14 days. Flow cytometry was performed to evaluate the phenotypes and functions of γδ T cells.

**Results:**

The proliferation of the γδ T cells was increased when PBMCs were cocultured with inactivated herpes virus entry mediator (HVEM)^low^ Jurkat cells. The cytotoxicity of the expanded γδ T cells was not affected by coculture with inactivated HVEM^low^ Jurkat cells and was further increased in the presence of anti‐PD‐L1 mAb. These results suggest that the inactivation of the BTLA signaling pathway during expansion could help produce more γδ T cells without compromising γδ T cell function. The inhibition of BTLA or PD‐1 signaling repressed phosphorylation of the src homology region 2‐containing protein tyrosine phosphatase 2 and increased the phosphorylation of protein kinase B in γδ T cells. However, there were no synergistic or additive effects by a combination of BTLA and PD‐1 blockade.

**Conclusion:**

These results suggest that BTLA signaling is crucial in regulating γδ T cell proliferation and function and that the BTLA and PD‐1 signaling pathways act independently on the proliferation and cytotoxicity of human peripheral γδ T cells.

## Graphical abstract

Blocking B‐ and T‐lymphocyte attenuator (BTLA) signaling increases γδ T cell proliferation ex vivo. BTLA signaling regulates γδ T cell proliferation and programmed cell death‐1 regulates effector function without affecting the other.

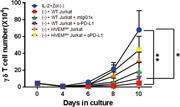

**Abbreviations**
AV: annexin V
BTLA: B‐ and T‐lymphocyte attenuator
CFSE: carboxyfluoroscein succinimidyl ester
FBS: fetal bovine serum
GVHD: graft versus host disease
HSCT: hematopoetic stem cell transplantation
HVEM: herpes virus entry mediator
IPP: isopentenyl pyrophosphates
ITIM: immunoreceptor tyrosine‐based inhibitory motif
ITSM: immunoreceptor tyrosine‐based switch motif
mAb: monoclonal antibody
NBP: aminobisphosphonate
PB: peripheral blood
PBMC: peripheral blood mononuclear cells
PBS: phosphate‐buffered saline
PD‐1: programmed cell death‐1
PD‐L1: PD‐ligand 1
PI: propidium iodide
SHP: src homology region 2 domain‐containing phosphatase
WT: wild‐type
Zol: zoledronate

## 1 INTRODUCTION

γδ T cells are innate‐like immune cells that participate in the early phase of infection and they kill tumor cells directly by degranulation of granzymes and perforin.^1^ γδ T cells constitute 1%–10% of total T cells. They have unique characteristics that distinguish them from αβ T cells, which make up the majority of T cells.^2^ Unlike αβ T cells, γδ T cells do not require MHC molecules for antigen recognition.^3^ Human Vγ9Vδ2 T cells, which are the majority of circulating γδ T cells, require cell‐to‐cell interactions to induce their reactivity. Vγ9Vδ2 T cells proliferate and are activated by recognizing phospho‐antigen directly.^1^

Through the mevalonate pathway, aminobisphosphonates (NBPs), such as zoledronate (Zol), lead to the accumulation of isopentenyl pyrophosphates (IPP) by inhibiting farnesyl‐pyrophosphate synthase. IPP is then recognized by a γδ T cell, which induces it to proliferate.^4^ The γδ T cells expanded and activated in response to IPP exert antitumor, antimicrobial, and antiviral responses.^5^, ^6^, ^7^ It has been reported that repeated and combined administration of NBPs and human Vγ9Vδ2 T cells can effectively control tumor growth in vivo.^8^ Human Vγ9Vδ2 T cells can directly kill leukemia cells^9^ without causing graft versus host disease (GVHD).^10^ It has been proven that the depletion of αβ T cells is a safe and effective tumor therapy.^11^ Clinical studies are underway to activate γδ T cells by administering Zol after αβ T and B cell‐depleted hematopoietic stem cell transplantation (HSCT) to obtain an optimal therapeutic effect based on the activity of γδ T cells.^12^, ^13^ Thus, γδ T‐cell adoptive transfer after HSCT could be an attractive therapeutic option.^14^, ^15^ It is, however, necessary to optimize methods of efficiently triggering the proliferation and activation of Vγ9Vδ2 T cells.

Coinhibitory or immune checkpoint signaling is one of the regulatory mechanisms to inhibit excessive T cell activation.^16^ However, it also causes tumor immune escape. Programmed cell death‐1 (PD‐1), an immune checkpoint molecule, reacts with programmed death‐ligand 1 (PD‐L1) to inhibit T cell activation, resulting in reduced cytotoxicity.^17^, ^18^ PD‐1 is highly expressed in exhausted T cells and is associated with impaired effector function and increased apoptosis.^19^, ^20^, ^21^ Increasing the antitumor response of T cells by inhibiting PD‐1 signaling has emerged as a promising therapy and its efficacy has been confirmed in preclinical and clinical trials.^22^, ^23^ B‐ and T‐lymphocyte attenuator (BTLA), a member of the CD28 family, negatively regulates T cell activation as does PD‐1.^24^ Both BTLA and PD‐1 contain Ig‐like domains that are expressed in T cells and B cells and present as monomers in T cells.^25^ After TCR stimulation, the immunoreceptor tyrosine‐based inhibitory motif (ITIM) and immunoreceptor tyrosine‐based switch motif (ITSM) of PD‐1 and BTLA are phosphorylated. Then, the src homology region 2 domain‐containing phosphatase (SHP)‐1 and SHP‐2 are recruited.^26^ As a result, downstream molecules of TCR and CD28 are dephosphorylated.^27^ Although PD‐1 and BTLA both have ITIM and ITSM motifs, PD‐1 essentially only requires ITSM for its inhibitory function,^28^ whereas BTLA requires both ITIM and ITSM.^26^ CD160, BTLA, and LIGHT share their common ligand, herpes virus entry mediator (HVEM), but BTLA is expressed almost exclusively in T cells.^29^ Our previous results showed that BTLA is robustly expressed on γδ T cells from leukemia patients.^30^ Blocking the BTLA/HVEM inhibitory signal improves the proliferation of γδ T cells.^29^ BTLA regulates the proliferation of Vγ9Vδ2 T cells,^29^, ^31^ whereas PD‐1 regulates their cytotoxicity.^32^

However, the interaction between the two inhibitory signaling pathways in Vγ9Vδ2 T cells is poorly understood. Therefore, we examined whether the BTLA and PD‐1 signaling pathways are convergent or independent in γδ T cells by evaluating the proliferation, cytotoxicity, and expression of signaling molecules. The results showed that the BTLA and PD‐1 signaling independently regulate the proliferation and activation of γδ T cells, suggesting that inhibition of BTLA signaling could improve the expansion of γδ T cells without compromising cytotoxicity.

## 2 MATERIALS AND METHODS

### 2.1 PBMC isolation and γδ T cell proliferation

Peripheral blood mononuclear cells (PBMC) or αβ T cell‐depleted PBMCs from healthy adult volunteers were isolated by density gradient centrifugation using Ficoll‐Paque™ Plus (GE Healthcare). αβ T cell‐depleted PBMCs were isolated using CliniMACS (Miltenyi Biotec), as previously described.^13^ All of the participants provided written informed consent. All of the procedures were approved by the Institutional Review Board, Asan Medical Center, Seoul, Korea (Approval No. 2015‐0307). The study was performed ethically, following the Declaration of Helsinki. Isolated cells were immediately frozen in heat‐inactivated fetal bovine serum (FBS) (Sigma‐Aldrich) containing 10% dimethyl sulfoxide (Sigma‐Aldrich) and were maintained in liquid nitrogen until use. The PBMCs were cultured at 6 × 10^5^ cells/well in 24‐well plates in Rosewell Park Memorial Institute (RPMI) 1640 (Corning) supplemented with 10% heat‐inactivated FBS, 100 IU/ml penicillin plus 100 μg/ml streptomycin (Corning), 1 mM sodium pyruvate (Sigma), and 55 μM 2‐mercaptoethanol (Gibco) in the presence of 100 U/ml recombinant human interleukin‐2 (IL‐2) (PeproTech) and 1 μM Zol (Selleckchem). Every 2 days, fresh IL‐2 was added.

### 2.2 Generation of HVEM^low^ Jurkat cell lines

Jurkat cells (ATCC) were cotransfected with the HVEM CRISPR/Cas9 KO plasmid (Santa Cruz) and the HVEM HDR plasmid (Santa Cruz) according to the manufacturer's instructions. After 48 h, all transfected cells were selected with media containing 2 μg/ml puromycin (TOCRIS Bioscience) for at least 2 weeks. Before use, the reduction of HVEM expression in the selected cells was confirmed by flow cytometry. The cells expressing lower levels of HVEM were named HVEM^low^ Jurkat cells. Jurkat cell lines were maintained in RPMI 1640 supplemented with 10% inactivated FBS, 100 IU penicillin plus 100 μg/ml streptomycin, and 1 mM sodium pyruvate. All cells were incubated in a humidified CO_2_ incubator.

### 2.3 Cell proliferation assay

PBMCs were washed and resuspended in phosphate‐buffered saline (PBS) (Biosesang). Then, the cells were labeled with cell proliferation Dye eFluor™ 670 (EF670) (Thermo Fisher Scientific) according to the manufacturer's instructions.

For inactivation of the Jurkat cells, they were treated with 25 μg/ml mitomycin C (Sigma) for 2 h and then it was washed away three times with 1× PBS. EF670‐labeled PBMCs at 3 × 10^5^ cells were cultured in 96‐well plates with or without the indicated monoclonal antibody and mitomycin C‐treated Jurkat cells at 1.5 × 10^5^ cells in the presence of 100 U/ml rhIL‐2 and 1 μM Zol. Anti‐human PD‐L1 blocking antibody (clone 29E.2A3; Biolegend) was added at 1 μg/ml at the beginning of the culture. Fresh IL‐2 was added every 2 days. In some experiments, BLTA/HVEM blocking peptides (Ac‐YRVKEACGELTGTVCEP‐NH_2_; Peptron),^33^ and anti‐PD‐1 mAb (clone EH12.2H7; Biolegend) were used. As control, scrambled peptide (Ac‐ELCAGPVTRKVECTYGE‐NH_2_; Peptron) and isotype control (clone MOPC‐21; Biolegend) were also used. The cultured cells were treated with FcR blocking reagent (Miltenyi Biotec) for 5 min and stained with phycoerythrin (PE)‐conjugated anti‐human TCRγδ (clone MOPC‐21; Biolegend) and APC‐H7‐conjugated anti‐human CD3 antibody (clone SK7; BD Biosciences) for 30 min. The EF670 dilution was analyzed by FACS Canto Ⅱ (BD Biosciences) or CytoFLEX (Beckman Coulter Life Sciences) flow cytometers and FlowJo (Tree Star, Inc.) software.

### 2.4 Degranulation assay

In the presence of anti‐human CD107a (clone eBioH4A3; eBioscience) monoclonal antibody, ex vivo expanded PBMC were incubated with tumor cells at a 1:1 ratio of effector:target. After 1 h, 50 μM monensin (eBioscience) was added. Following overnight incubation and washing, the cells were treated with FcR blocking reagent for 5 min, and stained with fluorescein isothiocyanate (FTIC)‐conjugated anti‐human TCRγδ (clone B1.1; eBioscience) and APC‐H7‐conjugated anti‐human CD3 antibody (clone SK7; BD Biosciences), then analyzed by FACS Canto Ⅱ or CytoFLEX flow cytometers and FlowJo software (Tree Star).

### 2.5 Cell death assay

Cytotoxicity was analyzed using annexin V (AV) and propidium iodide (PI) assays. Target cells were washed and resuspended in PBS. Then, the cells were labeled with carboxyfluorescein succinimidyl ester (CFSE) by using Cell Trace Cell Proliferation Kits (Molecular Probes) according to the manufacturer's instructions. Expanded γδ T cells were resuspended in media and incubated with CFSE‐labeled target cells. After 1 day, the cells were washed with AV‐binding buffer (BD Biosciences). The cells were treated with FcR blocking reagent for 5 min and stained with anti‐AV‐APC antibody (BD Biosciences) and PI (Biolegend). Early and late apoptosis was evaluated within the CFSE positive target cells by flow cytometry with FACS Canto Ⅱ or CytoFLEX. The data were analyzed by FlowJo software.

### 2.6 Flow cytometry

In vitro expanded γδ T cells were harvested and stained with the following antibodies: PE‐conjugated anti‐human TCRγδ (clone MOPC‐21; Biolegend), APC‐H7‐conjugated anti‐human CD3 antibody (clone SK7; BD Biosciences), FITC‐conjugated anti‐human CD279 antibody (clone EH12.2H7; Biolegend), and APC‐conjugated anti‐human CD272 antibody (clone MIH26; Biolegend). For analysis of the differentiation subsets, the following antibodies were used: FITC‐conjugated anti‐human TCRγδ (clone B1.1; eBioscience), APC‐H7‐conjugated anti‐human CD3 antibody (clone SK7; BD Biosciences), PE‐conjugated anti‐human CD45RA antibody (clone HI100; Biolegend), and APC‐conjugated anti‐human CD27 antibody (clone O323; eBioscience). Differentiation subsets were determined by CD45RA and CD27 expression within the γδ T cells. In some experiments, interferon‐γ (IFN‐γ) (clone 4S.B3; Biolegend) was intracellularly stained using BD Cytofix/Cytoperm™ (BD Biosciences). Data were acquired by flow cytometry. Flow cytometry was conducted by FACS Canto Ⅱ or CytoFLEX. The data were analyzed by FlowJo software.

### 2.7 Detection of phosphoprotein by flow cytometry

Jurkat cells were preincubated with anti‐human PD‐L1 blocking antibody (Biolegend) for 30 min in the presence of FcR blocking Ab and then treated with 100 μM pervanadate, an inhibitor of phosphatases, which was used as a phosphorylation inducer.^34^ In the presence of IL‐2 and Zol, PBMC and tumor cells were coincubated for 20 min and immediately fixed in a 4% paraformaldehyde solution. After washing with PBS containing 2% FBS, the cells were permeabilized with BD™ Phosflow Perm Buffer Ⅲ (BD Biosciences) for 30 min on ice. Then, they were incubated with anti‐pSHP‐2 (clone L99‐921; BD Biosciences), anti‐pAKT (Clone M89‐61; BD Biosciences), anti‐pERK1/2 (ab212153; Abcam), anti‐pSHP‐1 (ab192669; Abcam) antibody or IgG1κ isotype control for 1 h, following FcR blocking. For pSHP‐1 staining, PE‐conjugated goat antirabbit IgG Ab (ab72465; Abcam) was treated. Thirty minutes before washing the cells, FTIC‐conjugated TCRγδ (clone B1.1, eBioscience) and APC‐H7‐conjugated CD3 antibody (clone SK7; BD Biosciences) were additionally added. Flow cytometry was conducted by CytoFLEX (Beckman Coulter Life Sciences). The data were analyzed by FlowJo (Tree Star, Inc.) software to understand the phosphorylation status of SHP‐1/2, ERK1/2, and AKT.

### 2.8 Statistical analyses

Growth curves were analyzed by Friedman test, nonparametric repeated‐measures analysis of variance using InStat (GraphPad Software). Other results were analyzed using paired two‐tailed *t* tests by GraphPad Prism Ver. 6.0 software (GraphPad Software). When the *p* value was less than .05, the difference was considered to be significant.

## 3 RESULTS

### 3.1 BTLA signaling plays a critical role in the proliferation of γδ T cells

**Figure 1:**
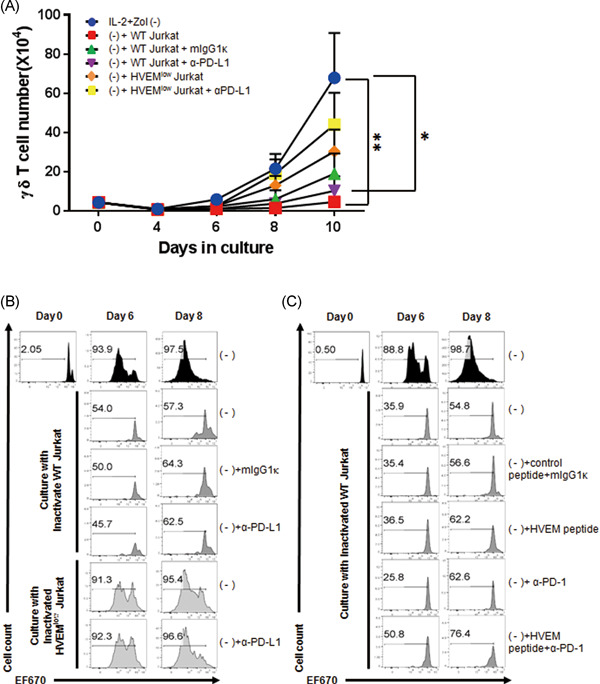
Blocking BTLA/HVEM interactions increase γδ T cell proliferation. (A) αβ T cell‐depleted PBMCs were cultured with inactivated Jurkat cells with or without anti‐human PD‐L1 blocking antibodies in the presence of IL‐2 and zoledronate(−). The gating strategy is shown in Figure S2. The frequency of γδ T cells (CD3^+^TCRγδ^+^) was determined by flow cytometry. The absolute numbers of γδ T cells (CD3^+^TCRγδ^+^) was calculated at the indicated days. Data were shown as mean ± SEM of six independent experiments (donor no. *n* = 6, except *n* = 3 for isotype control group). Friedman test was performed (*p < .05). (B) After EF670‐labeling, PBMCs were stimulated as described above. The top plots were only treated with IL‐2 and zoledronate(−) (Zol) and the lower plots were cultured with inactivated leukemic cells as indicated on the left. Cells were harvested at 6 or 8 days after culture, and the proliferation was determined by EF670 dilution by flow cytometry. Representative histograms are shown out of three independent experiments. (C) Experiment was performed as above, except that BTLA/HVEM blocking peptides and anti‐PD‐1 mAb were used instead of HVEM^low^ Jurkat cells and anti‐PD‐L1 Ab(−), IL‐2‐ and Zol‐treated. BTLA, B‐ and T‐lymphocyte attenuator; HVEM, herpes virus entry mediator; IL‐2, interleukin‐2; mAB, monoclonal antibody; mIgG1k, isotype control; PBMC, peripheral blood mononuclear cell; PD‐1, programmed cell death‐1; PD‐L1, programmed death‐ligand 1

We found that the proliferation of γδ T cells in the peripheral blood (PB) from healthy donors induced by IL‐2 and Zol was significantly impaired by coincubation with mitomycin C‐inactivated wild‐type (WT) Jurkat cells, which express HVEM (Figures 1A and S1). The differences between the control group, which was treated only with IL‐2 and Zol (IL‐2 + Zol(−)), and the groups coincubated with WT Jurkat in the absence or presence of anti‐PD‐L1 were statistically significant at Day 10. To determine whether blocking BTLA and/or PD‐1 signaling could improve the proliferation of the γδ T cells, PBMC were cultured with HVEM^low^ or WT Jurkat cells in the presence or absence of anti‐PD‐L1 blocking monoclonal antibody (mAb). The HVEM^low^ Jurkat cells were produced using a CRISPR/Cas9 system to delete the *HVEM* gene. As expected, HVEM expression was downregulated in HVEM^low^ Jurkat cells relative to mock‐transfected WT Jurkat cells (Figure S1). The expression of PD‐L1 was not changed by the deletion of *HVEM* in Jurkat cells (Figure S1, lower panel).

The inhibited proliferation of the γδ T cells in coculture with WT Jurkat cells was rescued by HVEM^low^ Jurkat cells (Figure 1A; *p* = .065). The gating strategy to identify γδ T cells was depicted in Figure S2. HVEM^low^ HL‐60 cells also increased γδ T cell proliferation, compared with WT HL‐60 cells (Figure S3A,B). Blocking PD‐1/PD‐L1 signaling increased the number of γδ T cells only in the presence of HVEM^low^ Jurkat cells. The treatment with anti‐PD‐L1 mAb alone failed to increase γδ T cell proliferation when BTLA/HVEM signaling was intact. These results were confirmed by EF670 dilution, as seen in Figure 1B. By Day 8, EF670^low/−^ proliferating cells were 97.5% when treated only with IL‐2 and Zol. Deletion of *HVEM* in Jurkat cells rescued the proliferating cells over 95% at Day 8, compared with 57.3% when cultured with WT Jurkat cells. The averages of the results were summarized in Figure S4. The treatment of BLTA/HVEM blocking peptides during culture increased γδ T cell proliferation which was inhibited by WT Jurkat cells, and anti‐PD‐1 Ab alone did not increase it (Figure 1C). Taken together, these results suggest that reduction of BTLA/HVEM signaling is a prerequisite for the optimal proliferation of Vδ2 γδ T cells, whereas PD‐1/PD‐L1 signaling has a limited role in their proliferation.

**Figure 2:**
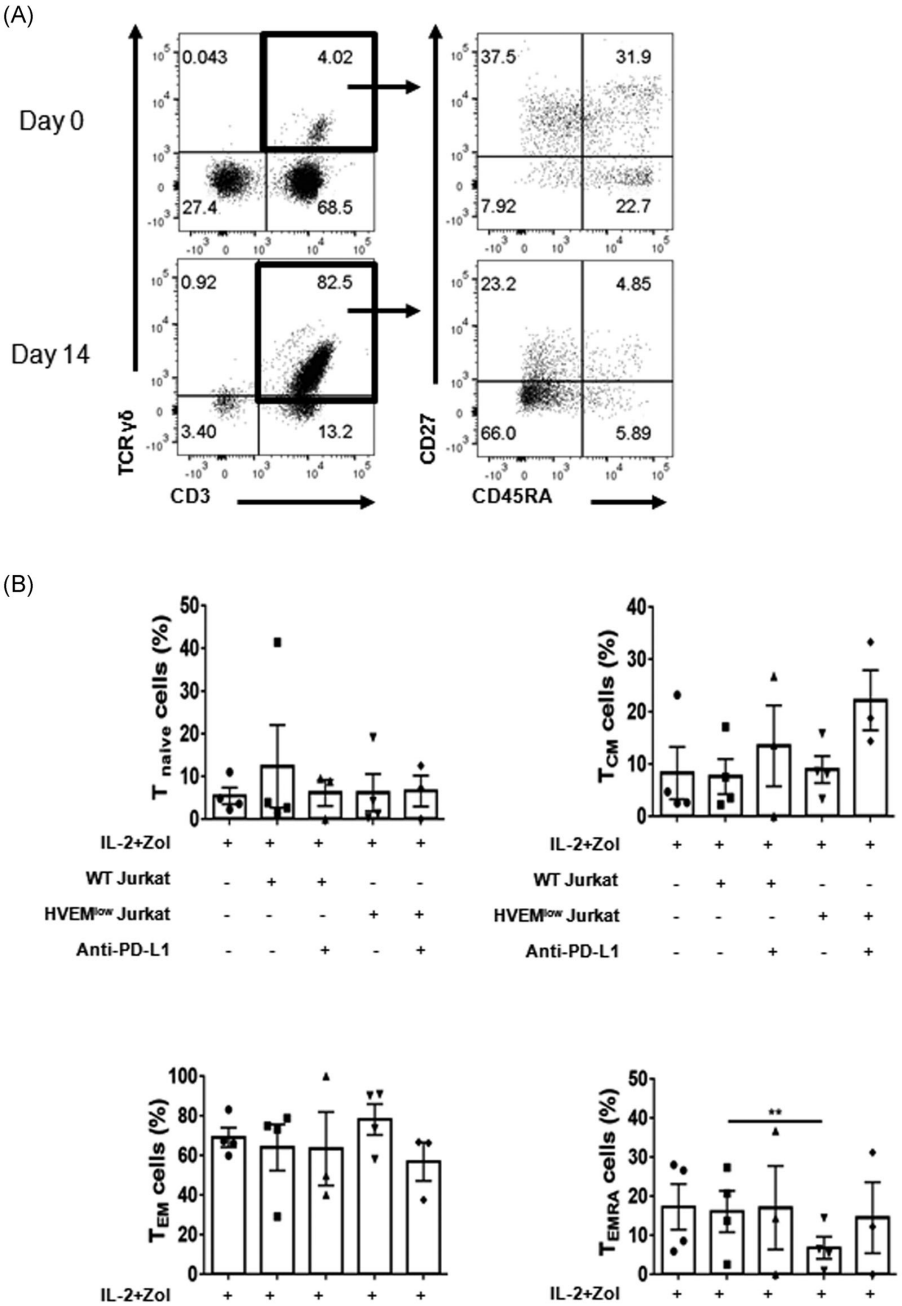
Blocking the BTLA/HVEM interaction reduces the terminally differentiated γδ T cell subset. (A) PBMC from healthy donors expanded in the presence of IL‐2 and zoledronate (Zol) for 14 days. Representative dot plots showing the phenotype of baseline or in vitro expanded cells (left panels) and the CD45RA and CD27 expression of the γδ T (CD3^+^TCRγδ^+^) cells (right panels). Representative data are shown out of three to four independent experiments using distinct donors. (B) PBMC were activated in the indicated culture condition for 14 days. The frequency of differentiation subsets in the in vitro expanded γδ T cells. The following subtypes were used for analysis: T naive (CD45RA^+^CD27^+^), T_CM_ (CD45RA^−^CD27^+^), T_EM_ (CD45RA^−^CD27^−^), and T_EMRA_ (CD45RA^+^CD27^−^). Data were shown as mean ± SEM of three to four independent experiments using distinct donors per groups. BTLA, B‐ and T‐lymphocyte attenuator; HVEM, herpes virus entry mediator; IL‐2, interleukin‐2; PBMC, peripheral blood mononuclear cell; T_CM_, central memory T; T_EM_, effector memory T; T_EMRA_, terminally differentiated T. Paired *t* test was used (**p < .01 and *p* = .0093)

**Figure 3:**
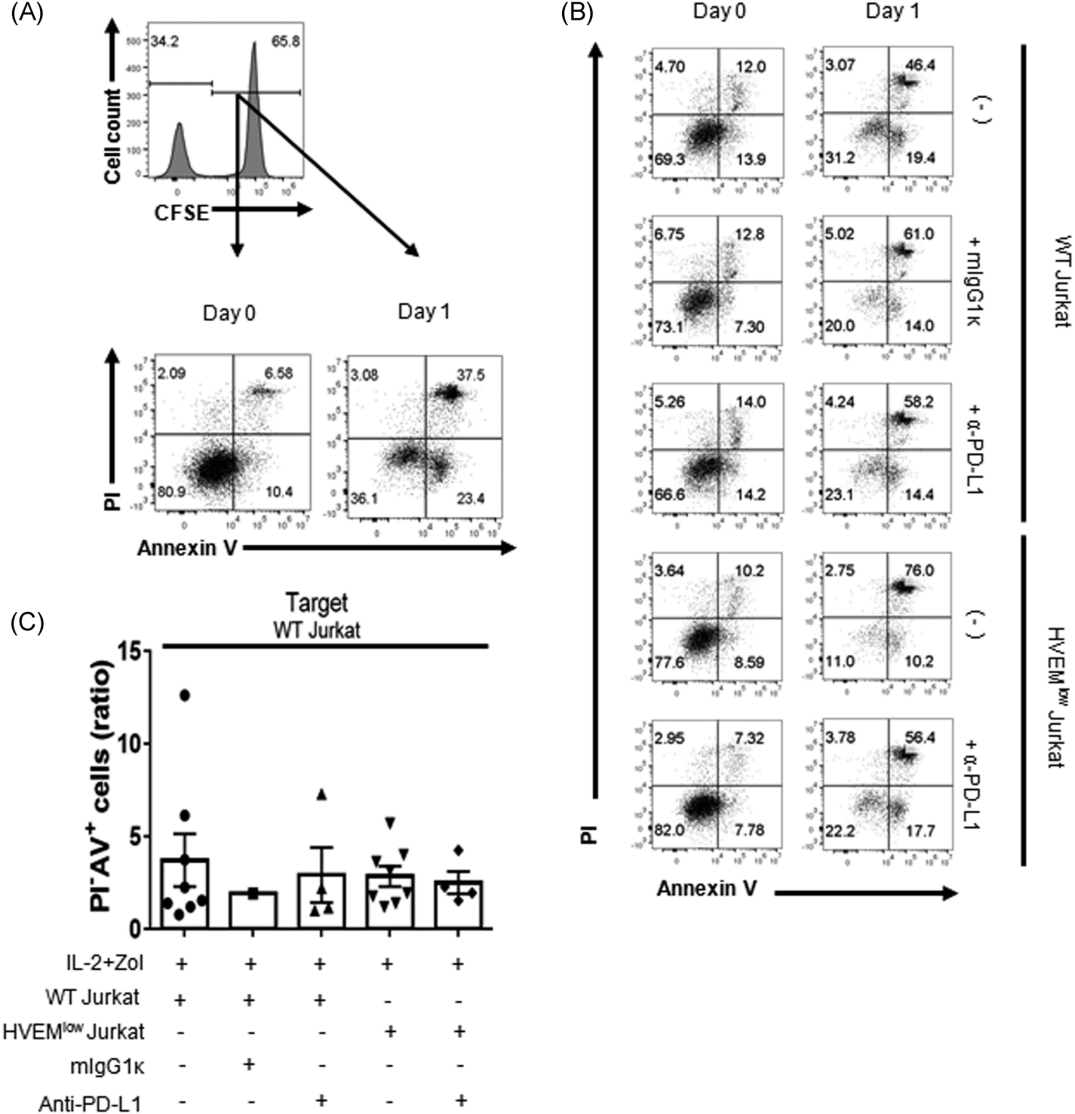
The targeted cell death by in vitro expanded γδ T cells was not altered by the blockade of BTLA/HVEM interaction. WT Jurkat cells were stained with CFSE and incubated with in vitro expanded γδ T cells at an effector to target cell ratio of 1:1 for 1 day. Cells were harvested and stained with PI and annexin V to detect apoptosis by flow cytometry. (A) Gating strategy to evaluate the PI and annexin V positive cells among the target cells. (B) Representative dot plots showing the proportion of early apoptosis (PI^−^Annexin V^+^) and late apoptosis (PI^+^Annexin V^+^) in the CFSE^+^ target cells. Left panel indicates the baseline (Day 0) and the right panel indicates after 1 day of incubation Representative data are shown out of three to seven independent experiments using distinct donors. (C) The percentage of PI^−^Annexin V^+^ apoptotic target cells in the experimental group were normalized by dividing by that of the control group Data were shown as mean ± SEM of three to seven independent experiments (donor no. *n* = 3–7). BTLA, B‐ and T‐lymphocyte attenuator; CFSE, carboxyfluorescein succinimidyl ester; HVEM, herpes virus entry mediator; mIgG1k, isotype control; PI, propidium iodide; WT, wild‐type. Paired *t* test was used (*p < .05 and *p* = .0471)

**Figure 4:**
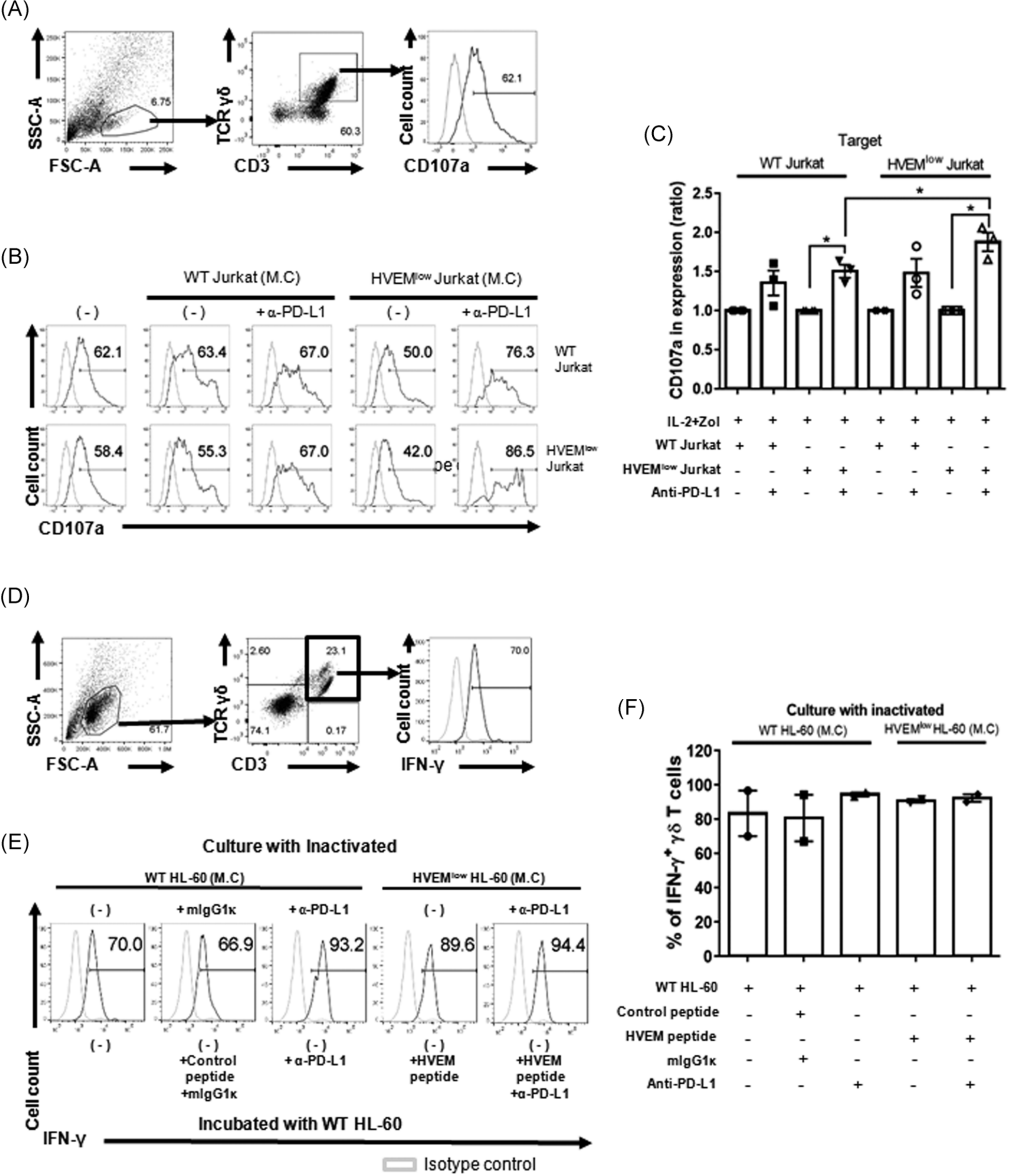
Blockade of the BTLA/HVEM signaling does not affect the degranulation of in vitro expanded γδ T cells. (A) Representative histograms showing CD107a expression in the γδ T cells. Gray lines indicate the isotype control. (B) Cells were expanded in the indicated culture condition for 14 days. In the presence of anti‐human CD107a mAb, in vitro expanded γδ T cells with the indicated inactivated cells (on top) were incubated overnight with WT Jurkat (upper panel) or HVEM^low^ Jurkat (lower panel) cells. Representative data are shown out of three to four independent experiments using distinct donors. (C) The percentages of CD107a^+^ γδ T cells in the anti‐PD‐L1 treated group were normalized by dividing by that of the untreated group. Data were shown as mean ± SEM of three to four independent experiments using distinct donors. Paired *t* test was used (*p < .05). Each *P* value is .0190, .0345, and .0103 from left to right. (D) The gating strategy to identify intracellular IFN‐γ is depicted. (E) Expanded γδ T cells with the indicated inactivated cells (on top) were incubated overnight with WT Jurkat cells in the presence of BTLA/HVEM blocking peptides and anti‐PD‐L1 Ab or adequate controls for IFN‐γ intracellular staining. (F) The percentages of IFN‐γ^+^ γδ T cells were summarized. *N* = 2. BTLA, B‐ and T‐lymphocyte attenuator; HVEM, herpes virus entry mediator; IFN‐γ, interferon; mAB, monoclonal antibody; PD‐L1, programmed death‐ligand 1; WT, wild‐type

PD‐1 expression on γδ T cells was assessed to determine whether the reduction of BTLA/HVEM or PD‐1/PD‐L1 signaling during the ex vivo expansion altered PD‐1 expression on the γδ T cells. Stimulation with IL‐2 and Zol for 10 days significantly upregulated BTLA and PD‐1 expression on the γδ T cells, and reduction of BTLA and PD‐1 signaling did not change BTLA and PD‐1 expression on the γδ T cells (Figure S5).

**Figure 5:**
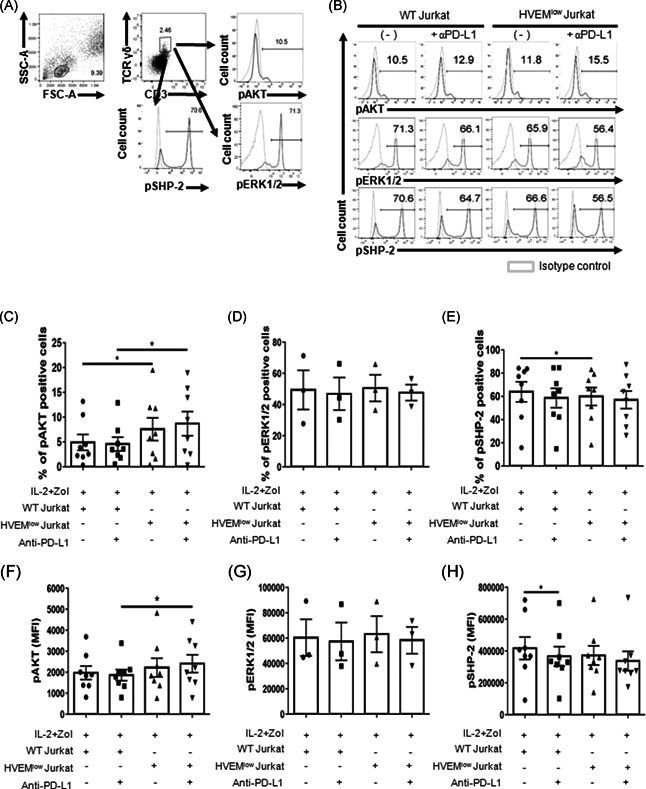
BTLA/HVEM and PD‐1/PD‐L1 signals do not additively activate the downstream molecules. Phosphorylation of AKT, ERK1/2, and SHP‐2 in γδ T cells was measured by flow cytometry. WT or HVEM^low^ Jurkat cells were pretreated with anti‐human PD‐L1 antibody for 30 min. In the presence of pervanadate, PBMCs were stimulated with IL‐2 and zoledronate and coincubated with indicated cells for 20 min. After fixation/permeabilization by PFA and methanol, the cells were stained with anti‐human CD3, TCRγδ, phospho‐AKT, ERK, and SHP‐2 Abs. (A) The gating strategy was depicted. (B) Representative histograms are shown. (C, F) The percentages and mean fluorescence indices of pAKT. Data were shown as mean ± SEM of eight to nine independent experiments. (D, G). The percentages and mean fluorescence indices of pERK1/2. Data were shown as mean ± SEM of three independent experiments. (E, H) The percentages and mean fluorescence indices of pSHP‐2. Data were shown as mean ± SEM of eight to nine independent experiments. BTLA, B‐ and T‐lymphocyte attenuator; HVEM, herpes virus entry mediator; IL‐2, interleukin‐2; PBMC, peripheral blood mononuclear cell; PD‐1, programmed cell death‐1; PD‐L1, programmed death‐ligand 1; SHP, src homology region 2 domain‐containing phosphatase; WT, wild‐type. Paired *t* test was used (*p < .05). Individual *p* values as below: (C) *p* = .0384 and 0.0311 from left to right; (E) *p* = .0480; (F) *p* = .0307; (H) *p* = .0377

### 3.2 Blocking BTLA signaling reduces the more differentiated γδ T cell population

It has been suggested that the more differentiated T cells are, the less antitumor efficacy they have,^35^ and thus the subsets of expanded γδ T cells were analyzed by flow cytometry. As seen in Figure 2, coincubation with HVEM^low^ Jurkat cells reduced the proportion of terminally differentiated CD27^−^CD45RA^+^ γδ T cells significantly, compared with that incubated with WT Jurkat cells. Coincubation with WT Jurkat cells slightly increased the number of CD27^+^CD45RA^+^ naïve T cells, and coincubation with HVEM^low^ Jurkat cells in the presence of PD‐L1 blockade increased the number of CD27^+^CD45RA^−^ central memory T cells, although the differences were not statistically significant. These results imply that inhibiting BTLA signaling pathway during expansion could be beneficial for cancer immunotherapy by preventing terminal differentiation of γδ T cells.

### 3.3 The antitumor activity of γδ T cells is not affected by blocking BTLA signaling

To evaluate the effect of a blockade of BTLA/HVEM and PD‐1/PD‐L1 signaling on the effector function of γδ T cells, PBMCs were treated with anti‐human PD‐L1 mAb while cocultured with HVEM^low^ Jurkat or WT Jurkat cells in the presence of IL‐2 and Zol. We then examined the antitumor activity of ex vivo expanded γδ T cells using PI and AV in tumor cells. The cells expanded under the conditions shown in Figure 4 were incubated with the same number of CFSE‐labeled Jurkat cells for 1 day (Figure 3A). PBMC proliferated by IL‐2 and Zol treatment effectively induced apoptosis in all Jurkat cell lines (Figure 3B,C). Likewise, γδ T cells cultured with WT Jurkat cells or HVEM^low^ Jurkat cells caused apoptosis of the target cells comparable to the PBMC control group. Contrary to expectations, anti‐PD‐L1 mAb treatment did not increase both the early and late apoptosis of the target cells, despite increased expression of CD107a (Figure 4).

The expanded γδ T cells were challenged by HVEM^low^ Jurkat or WT Jurkat cells, and then, CD107a expression was measured to detect the degranulation of the ex vivo expanded γδ T cells. Ex vivo expanded γδ T cells in all five groups expressed similar levels of CD107a in response to HVEM^low^ Jurkat and WT Jurkat cells. Inhibitory signaling by the WT and the HVEM^low^ Jurkat cells did not affect the degranulation of γδ T. However, the expression of CD107a on γδ T cells cocultured with HVEM^low^ Jurkat cells was significantly upregulated by the additional inhibition of PD‐L1 signaling. Taken together, PD‐L1 signaling, rather than HVEM signaling, regulated the degranulation of γδ T cells. The experiments with HVEM^low^ HL‐60 cells also had no significant effect on degranulation (Figure S6). Collectively, the reduced HVEM signaling did not alter the target cell death by γδ T cells during the culture as well as the reaction period.

γδ T cells could produce cytokines, such as IFN‐γ, upon stimulation. Thus we assessed intracellular IFN‐γ by flow cytometry (Figure 4D,E). γδ T cells were cultured with inactivated WT or HVEM^low^ Jurkat cells in the presence or absence of anti‐PD‐L1 Ab for 2 weeks, and the cells were stimulated with WT Jurkat cells in the presence of BTLA/HVEM blocking peptides and/or anti‐PD‐L1 Ab to measure intracellular IFN‐γ. Blocking PD‐1 signaling clearly increased IFN‐γ production, whereas that of BTLA had only minor effects. In summary, the reduction of BTLA signaling during expansion did not affect the function of γδ T cells.

### 3.4 BTLA and PD‐1 signaling are independent of each other in γδ T cells

BTLA and PD‐1 control cell proliferation and apoptosis through downstream molecules, such as AKT, ERK, and SHP‐2, which mediate T cell activating and inhibitory signalings, respectively. Thus, we next investigated the effects of immediate BTLA/HVEM and PD‐1/PD‐L1 signaling on the phosphorylation of AKT, ERK, and SHP‐2 in resting γδ T cells. PBMCs were incubated with WT Jurkat or HVEM^low^ Jurkat cells and/or anti‐PD‐L1 mAb in the presence of IL‐2 and Zol for 20 min, and the expression of the phosphorylated protein was evaluated in γδ T cells by flow cytometry. We observed that the phosphorylation of AKT was increased slightly by blockade of the PD‐1/PD‐L1 interaction, and a reduction of BTLA/HVEM signaling in addition to it further increased pAKT, which was statistically significant in the percentages as well as MFI (Figures 5C and 5F). Likewise, coincubation with HVEM^low^ Jurkat cells also increased pAKT, compared with that of WT Jurkat cells. The activation of ERK was not changed in the presence of IL‐2 and Zol (Figures 5D and 5G). The induction of pSHP‐2 was slightly repressed by anti‐human PD‐L1 treatment in MFI, and inhibition of the BTLA/HVEM interaction reduced the percentage of pSHP‐2^+^ γδ T cells. However, BTLA/HVEM and PD‐1/PD‐L1 signaling had no synergistic or additive effects on the phosphorylation of SHP‐2 (Figures 5E and 5H).

## 4 DISCUSSION

Previous research on the individual signals of BTLA and PD‐1 in circulating γδ T cells has been conducted,^29^, ^32^ but there are few reports on the interactions of these signals. In this study, the effects of the BTLA/HVEM and PD‐1/PD‐L1 inhibitory signaling pathways were evaluated on the proliferation and effector functions of human peripheral γδ T cells, in particular Vγ9Vδ2 T cells. We found that BTLA/HVEM blockade could improve the efficient expansion of human peripheral γδ T cells without compromising γδ T cell function. These results could be utilized for the development of γδ T cell therapy for leukemia patients. In addition, the results suggest that the BTLA and PD‐1 signaling pathways independently act on the proliferation and cytotoxicity of γδ T cells.

First, we found that coculture with inactivated leukemia cells hampered the expansion of human peripheral γδ T cells severely. This may explain the insufficient elimination of tumor cells by γδ T cells in vivo in patients with leukemia, where BTLA on PB γδ T cells can come into contact with HVEM on leukemic cells. Coincubation with WT Jurkat cells slightly increased the proportion of CD27^+^CD45RA^+^ naïve T cells, which are known to be associated with GVHD.^36^ The proliferation capacity of γδ T cells was upregulated by blocking the HVEM/BTLA interaction without reducing their cytotoxicity, whereas inhibition of PD‐1/PD‐L1 signaling induced degranulation of γδ T cells. We chose to inhibit the BTLA and PD‐1 ligands on the target cells, rather than BTLA and PD‐1 directly because we were concerned that the direct blockade of BTLA and PD‐1 might unexpectedly affect the proliferation and function of γδ T cells during expansion. However, BTLA also binds to the HCMV UL144 protein to limit lymphocyte‐induced inflammation.^37^ Potential interactions of BTLA with other ligands might explain the partial inhibition of downstream molecules in this study. In addition, we cannot exclude the possibility that Fc receptors on the γδ T cells might play a stimulating role in response to anti‐PD‐L1 mAb. Thus, we confirmed the results with BTLA/HVEM blocking peptides and anti‐PD‐1 mAb and with HVEM^low^ HL‐60 cells, which also improved γδ T cell expansion.

In previous reports, both PD‐1 and BTLA inhibit the proliferation of T cells.^24^, ^38^ Moreover, BTLA negatively controls the homeostasis of γδ T cells^39^ and inhibits phospho‐antigen‐induced γδ T cell proliferation.^29^ Consistent with previous reports, HVEM‐deleted Jurkat cells enhanced the proliferation capacity of γδ T cells more than WT Jurkat cells. However, an anti‐PD‐L1 blockade alone does not have a proliferative effect on γδ T cells. Taken together, the expansion of γδ T cells is tightly regulated by BTLA rather than PD‐1.

It should be mentioned that the deletion of the *HVEM* gene did not affect endogenous PD‐L1 expression on Jurkat cells and HL‐60 cells and that blocking the BTLA and PD‐1 signaling pathways did not affect each other's expression on γδ T cells either. Although γδ T cells can transiently upregulate PD‐L1 expression,^40^ PD‐L1 do not have signaling domains in its short cytoplasmic tail.^41^ Thus, it is unlikely blocking PD‐L1 on γδ T cells could affect the results in this study. In one study, the expression of BTLA was downregulated by IL‐2 and bromohydrin pyrophosphate after 4 days, whereas the expression of PD‐1 was upregulated.^29^ However, we observed a significant increase in the expression of BTLA and PD‐1 in γδ T cells expanded with IL‐2 and Zol 10 days. It has previously been reported that the degranulation and cytotoxicity of PD‐1^+^ γδ T cells were reduced in response to PD‐L1‐expressing target cells, but they were recovered by anti‐PD‐L1 treatment.^32^ Similarly, we observed that inhibition of sustained PD‐1 signaling increased CD107a degranulation of γδ T cells. However, it was insufficient to cause enhanced cell death in the target cells.

PD‐1 upregulates the expression of SHP‐2 in tumor‐infiltrating lymphocytes,^42^ and inhibition of PD‐1 signaling suppresses the phosphorylation of SHP‐2.^43^ Likewise, pSHP‐2 was slightly less induced by inhibition of PD‐1 signaling against WT Jurkat cells in our system. In addition, SHP‐2 is associated with the phosphorylation of BTLA by TCR engagement.^24^ We observed that the specific inhibition of the BTLA/HVEM and PD‐1/PD‐L1 pathways in γδ T cells reduced phosphorylation of SHP‐2 in percentages or MFI, but the combined inhibition did not show any additional effect. Reduction of the BTLA/HVEM or PD‐1/PD‐L1 signal in γδ T cells inhibited the induction of pSHP‐2 in response to Jurkat cells, whereas phosphorylation of AKT was increased by the reduction of both BTLA/HVEM and PD‐1/PD‐L1 signals. As immune checkpoint receptors, BTLA and PD‐1 signaling appear to play independent roles in inhibiting γδ T cell proliferation and cytotoxicity, respectively, and they had no additive effect on each other. In a recent report, BTLA and PD‐1 regulate T cell signaling differentialy and only partially through SHP‐1 and SHP‐2,^44^ that supports our study. In Terms of activating signaling, dual inhibition of BTLA and PD‐1 increased pAKT, compared with that of BTLA single blockade, implying the importance of BTLA signaling in γδ T cells.

In conclusion, BTLA/HVEM inhibitory signaling is a crucial factor in proliferation in ex vivo expanded Vδ2 γδ T cells. On the contrary, PD‐1/PD‐L1 signaling could provide partial enhancement in proliferation and cytotoxicity. These results indicate the importance of BTLA signaling in γδ T cells and suggest that simultaneous suppression of both signals could effectively improve immunotherapy with γδ T cells. Furthermore, the results of this study could lead to a better understanding of the interactions between BTLA/HVEM and PD‐1/PD‐L1 signaling in tumor microenvironments.

## CONFLICT OF INTERESTS

The authors declare that there are no conflict of interests.

## AUTHOR CONTRIBUTIONS

Ho J. Im and Nayoung Kim conceived, designed, and discussed the research. Eun S. Choi, Sung H. Kang, and Kyung‐Nam Koh provided samples and analyzed the results. Hyun J. Hwang, Jae J. Lee, Seongsoo Jang, Sang‐Hyun Hwang, and Nayoung Kim conducted the research and analyzed the results. Kyung‐Nam Koh, Sang‐Hyun Hwang, and Nayoung Kim obtained the grants. Hyun J. Hwang and Nayoung Kim wrote the manuscript.

## Supporting information

Supporting information.

Supporting information.

## Data Availability

The data that support the findings of this study are available from the corresponding author upon reasonable request.

## References

[1] Chien YH , Meyer C , Bonneville M. gammadelta T cells: first line of defense and beyond. Annu Rev Immunol. 2014;32:121‐155. 24387714 10.1146/annurev-immunol-032713-120216

[2] Vantourout P , Hayday A. Six‐of‐the‐best: unique contributions of gammadelta T cells to immunology. Nat Rev Immunol. 2013;13:88‐100. 23348415 10.1038/nri3384 PMC3951794

[3] Kabelitz D , Glatzel A , Wesch D. Antigen recognition by human gammadelta T lymphocytes. Int Arch Allergy Immunol. 2000;122:1‐7. 10859464 10.1159/000024353

[4] Thurnher M , Nussbaumer O , Gruenbacher G. Novel aspects of mevalonate pathway inhibitors as antitumor agents. Clin Cancer Res. 2012;18:3524‐3531. 22529099 10.1158/1078-0432.CCR-12-0489

[5] Bonneville M , Scotet E. Human Vgamma9Vdelta2 T cells: promising new leads for immunotherapy of infections and tumors. Curr Opin Immunol. 2006;18:539‐546. 16870417 10.1016/j.coi.2006.07.002

[6] Qin G , Mao H , Zheng J , et al. Phosphoantigen‐expanded human gammadelta T cells display potent cytotoxicity against monocyte‐derived macrophages infected with human and avian influenza viruses. J Infect Dis. 2009;200:858‐865. 19656068 10.1086/605413 PMC7110194

[7] Xiang Z , Liu Y , Zheng J , et al. Targeted activation of human Vgamma9Vdelta2‐T cells controls epstein‐barr virus‐induced B cell lymphoproliferative disease. Cancer Cell. 2014;26:565‐576. 25220446 10.1016/j.ccr.2014.07.026

[8] Santolaria T , Robard M , Leger A , Catros V , Bonneville M , Scotet E. Repeated systemic administrations of both aminobisphosphonates and human Vgamma9Vdelta2 T cells efficiently control tumor development in vivo. J Immunol. 2013;191:1993‐2000. 23836057 10.4049/jimmunol.1300255

[9] Gertner‐Dardenne J , Castellano R , Mamessier E , et al. Human Vgamma9Vdelta2 T cells specifically recognize and kill acute myeloid leukemic blasts. J Immunol. 2012;188:4701‐4708. 22467661 10.4049/jimmunol.1103710

[10] Lamb LS, Jr. , Lopez RD . gammadelta T cells: a new frontier for immunotherapy? Biol Blood Marrow Transplant. 2005;11:161‐168. 15744234 10.1016/j.bbmt.2004.11.015

[11] Choi ES , Im HJ , Kim H , et al. Depletion of alphabeta(+) T cells for a haploidentical hematopoietic stem cell transplantation in children. J Clin Apher. 2018;33:521‐528. 29971847 10.1002/jca.21634

[12] Bertaina A , Zorzoli A , Petretto A , et al. Zoledronic acid boosts gammadelta T‐cell activity in children receiving alphabeta(+) T and CD19(+) cell‐depleted grafts from an HLA‐haplo‐identical donor. Oncoimmunology. 2017;6:e1216291. 28344861 10.1080/2162402X.2016.1216291 PMC5353934

[13] Koh KN , Im HJ , Kim H , et al. alphabeta T‐cell‐depleted haploidentical hematopoietic cell transplantation and zoledronate/interleukin‐2 therapy in children with relapsed, high‐risk neuroblastoma. Bone Marrow Transplant. 2019;54:348‐352. 30116016 10.1038/s41409-018-0305-3

[14] Handgretinger R , Schilbach K. The potential role of gammadelta T cells after allogeneic HCT for leukemia. Blood. 2018;131:1063‐1072. 29358176 10.1182/blood-2017-08-752162

[15] Sebestyen Z , Prinz I , Dechanet‐Merville J , Silva‐Santos B , Kuball J. Translating gammadelta (gammadelta) T cells and their receptors into cancer cell therapies. Nat Rev Drug Discovery. 2020;19:169‐184. 31492944 10.1038/s41573-019-0038-z

[16] Zhang Q , Vignali DA . Co‐stimulatory and co‐inhibitory pathways in autoimmunity. Immunity. 2016;44:1034‐1051. 27192568 10.1016/j.immuni.2016.04.017 PMC4873959

[17] Dong Y , Sun Q , Zhang X. PD‐1 and its ligands are important immune checkpoints in cancer. Oncotarget. 2017;8:2171‐2186. 27974689 10.18632/oncotarget.13895 PMC5356790

[18] Freeman GJ , Long AJ , Iwai Y , et al. Engagement of the PD‐1 immunoinhibitory receptor by a novel B7 family member leads to negative regulation of lymphocyte activation. J Exp Med. 2000;192:1027‐1034. 11015443 10.1084/jem.192.7.1027 PMC2193311

[19] Wherry EJ , Kurachi M. Molecular and cellular insights into T cell exhaustion. Nat Rev Immunol. 2015;15:486‐499. 26205583 10.1038/nri3862 PMC4889009

[20] Jiang Y , Li Y , Zhu B. T‐cell exhaustion in the tumor microenvironment. Cell Death Dis. 2015;6:e1792. 26086965 10.1038/cddis.2015.162 PMC4669840

[21] Pauken KE , Wherry EJ . SnapShot: T cell exhaustion. Cell. 2015;163:1038. 26544946 10.1016/j.cell.2015.10.054

[22] Sznol M , Chen L. Antagonist antibodies to PD‐1 and B7‐H1 (PD‐L1) in the treatment of advanced human cancer. Clin Cancer Res. 2013;19:1021‐1034. 23460533 10.1158/1078-0432.CCR-12-2063 PMC3702373

[23] Nguyen LT , Ohashi PS . Clinical blockade of PD1 and LAG3—potential mechanisms of action. Nat Rev Immunol. 2015;15:45‐56. 25534622 10.1038/nri3790

[24] Watanabe N , Gavrieli M , Sedy JR , et al. BTLA is a lymphocyte inhibitory receptor with similarities to CTLA‐4 and PD‐1. Nat Immunol. 2003;4:670‐679. 12796776 10.1038/ni944

[25] Chemnitz JM , Lanfranco AR , Braunstein I , Riley JL . B and T lymphocyte attenuator‐mediated signal transduction provides a potent inhibitory signal to primary human CD4 T cells that can be initiated by multiple phosphotyrosine motifs. J Immunol. 2006;176:6603‐6614. 16709818 10.4049/jimmunol.176.11.6603

[26] Gavrieli M , Watanabe N , Loftin SK , Murphy TL , Murphy KM . Characterization of phosphotyrosine binding motifs in the cytoplasmic domain of B and T lymphocyte attenuator required for association with protein tyrosine phosphatases SHP‐1 and SHP‐2. Biochem Biophys Res Commun. 2003;312:1236‐1243. 14652006 10.1016/j.bbrc.2003.11.070

[27] Chen L , Flies DB . Molecular mechanisms of T cell co‐stimulation and co‐inhibition. Nat Rev Immunol. 2013;13:227‐242. 23470321 10.1038/nri3405 PMC3786574

[28] Chemnitz JM , Parry RV , Nichols KE , June CH , Riley JL . SHP‐1 and SHP‐2 associate with immunoreceptor tyrosine‐based switch motif of programmed death 1 upon primary human T cell stimulation, but only receptor ligation prevents T cell activation. J Immunol. 2004;173:945‐954. 15240681 10.4049/jimmunol.173.2.945

[29] Gertner‐Dardenne J , Fauriat C , Orlanducci F , et al. The co‐receptor BTLA negatively regulates human Vgamma9Vdelta2 T‐cell proliferation: a potential way of immune escape for lymphoma cells. Blood. 2013;122:922‐931. 23692853 10.1182/blood-2012-11-464685

[30] Kang SH , Hwang HJ , Yoo JW , et al. Expression of immune checkpoint receptors on T‐cells and their ligands on leukemia blasts in childhood acute leukemia. Anticancer Res. 2019;39:5531‐5539. 31570447 10.21873/anticanres.13746

[31] Gertner‐Dardenne J , Fauriat C , Olive D. BTLA, a key regulator of Vgamma9Vdelta2 T‐cell proliferation. Oncoimmunology. 2013;2:e25853. 24244908 10.4161/onci.25853 PMC3825728

[32] Iwasaki M , Tanaka Y , Kobayashi H , et al. Expression and function of PD‐1 in human gammadelta T cells that recognize phosphoantigens. Eur J Immunol. 2011;41:345‐355. 21268005 10.1002/eji.201040959

[33] Spodzieja M , Kuncewicz K , Sieradzan A , et al. Disulfide‐linked peptides for blocking BTLA/HVEM‐binding. Int J Mol Sci. 2020;21:8876. 10.3390/ijms21020636 PMC7013932 31963646

[34] Imbert V , Peyron JF , Farahi Far D , Mari B , Auberger P , Rossi B. Induction of tyrosine phosphorylation and T‐cell activation by vanadate peroxide, an inhibitor of protein tyrosine phosphatases. Biochem J. 1994;297(pt 1):163‐173. 7506531 10.1042/bj2970163 PMC1137806

[35] Restifo NP , Dudley ME , Rosenberg SA . Adoptive immunotherapy for cancer: harnessing the T cell response. Nat Rev Immunol. 2012;12:269‐281. 22437939 10.1038/nri3191 PMC6292222

[36] Triplett BM , Shook DR , Eldridge P , et al. Rapid memory T‐cell reconstitution recapitulating CD45RA‐depleted haploidentical transplant graft content in patients with hematologic malignancies. Bone Marrow Transplant. 2015;50:968‐977. 25665048 10.1038/bmt.2014.324 PMC4636007

[37] Sedy JR , Bjordahl RL , Bekiaris V , et al. CD160 activation by herpesvirus entry mediator augments inflammatory cytokine production and cytolytic function by NK cells. J Immunol. 2013;191:828‐836. 23761635 10.4049/jimmunol.1300894 PMC3702646

[38] Sheppard KA , Fitz LJ , Lee JM , et al. PD‐1 inhibits T‐cell receptor‐induced phosphorylation of the ZAP70/CD3zeta signalosome and downstream signaling to PKCtheta. FEBS Lett. 2004;574:37‐41. 15358536 10.1016/j.febslet.2004.07.083

[39] Bekiaris V , Sedy JR , Macauley MG , Rhode‐Kurnow A , Ware CF . The inhibitory receptor BTLA controls gammadelta T cell homeostasis and inflammatory responses. Immunity. 2013;39:1082‐1094. 24315996 10.1016/j.immuni.2013.10.017 PMC3909738

[40] Peters C , Oberg HH , Kabelitz D , Wesch D. Phenotype and regulation of immunosuppressive Vdelta2‐expressing gammadelta T cells. Cell Mol Life Sci. 2014;71:1943‐1960. 24091816 10.1007/s00018-013-1467-1 PMC3997799

[41] Akinleye A , Rasool Z. Immune checkpoint inhibitors of PD‐L1 as cancer therapeutics. J Hematol Oncol. 2019;12:92. 31488176 10.1186/s13045-019-0779-5 PMC6729004

[42] Li J , Jie HB , Lei Y , et al. PD‐1/SHP‐2 inhibits Tc1/Th1 phenotypic responses and the activation of T cells in the tumor microenvironment. Cancer Res. 2015;75:508‐518. 25480946 10.1158/0008-5472.CAN-14-1215 PMC4315704

[43] Yamamoto R , Nishikori M , Kitawaki T , et al. PD‐1‐PD‐1 ligand interaction contributes to immunosuppressive microenvironment of Hodgkin lymphoma. Blood. 2008;111:3220‐3224. 18203952 10.1182/blood-2007-05-085159

[44] Xu X , Hou B , Fulzele A , et al. PD‐1 and BTLA regulate T cell signaling differentially and only partially through SHP1 and SHP2. J Cell Biol. 2020;219:e201905085. 32437509 10.1083/jcb.201905085 PMC7265324

