# Supplementary material for: The BTLA and PD‐1 signaling pathways independently regulate the proliferation and cytotoxicity of human peripheral blood γδ T cells
Source: Immun Inflamm Dis. 2020 Dec 17;9(1):274–87. doi: 10.1002/iid3.390 (PMC7860523; doi:10.1002/iid3.390)
Supplement: Supplementary file 1 — Supporting information. [file IID3-9-274-s001.docx]

**Supporting Figure Legends**

**Supporting Figure 1. Deletion of HVEM does not affect endogenous PD-L1 expression in Jurkat cells.** Flow cytometric analysis of HVEM (upper panel) and the PD-L1 (lower panel) expression on WT Jurkat (Blue line) and HVEM^low^ Jurkat cells (Red line). The representative histograms are shown. The numerical values indicate the percentages of HVEM or PD-L1 positive cells among the HVEM^low^ Jurkat cells. Representative data are shown out of 3 independent experiments.

**Supporting Figure 2. Gating strategy to define γδ T cells.** The γδ T cells are defined as CD3^+^ γδTCR^+^.

**Supporting Figure 3, Blocking BTLA/HVEM interactions increases γδ T cell proliferation.** (A) Deletion of HVEM in HL-60 cells do not affect PD-1 expression. Flow cytometric analysis of HVEM (upper panel) and the PD-L1 (lower panel) expression on WT HL-60 (Blue line) and HVEM^low^ HL-60 cells (Red line). The Yellow lines indicate the isotype control. The representative histograms are shown. The numerical values indicate the percentages of HVEM or PD-L1 positive cells among the HVEM^low^ HL-60 cells. (B) PBMCs were cultured with inactivated WT or HVEM^low^ HL-60 cells in the presence of IL-2 and Zol (-). The frequency of γδ T cells (CD3^+^TCRγδ^+^) was determined by flow cytometry. The absolute number of γδ T cells (CD3^+^TCRγδ^+^) at 14 days after culture was divided by that of Day 0. Data shown as 2 independent experiments (Donr No. n=2). (-), IL-2- and Zol-treated; M.C., mitomycin C-treated.

**Supporting Figure 4. Summary of EF670 cell proliferation assay.** The results in Fig. 1B was summarized as means±SEM. Left panel is for Day 6 and Right Panel is for Day 8. n=2-3. Student *t* test was performed. **p*<0.05.

**Supporting Figure 5.** **Co-incubation with HVEM^low^ Jurkat cells does not affect BTLA or PD-1 expression on γδ T cells.** PBMCs were expanded by IL-2 and Zol for 10 days and stained with anti-human CD3, TCRγδ, BTLA, and PD-1 mAb. (A) Representative histograms showing BTLA and PD-1 expression on the γδ T cells on day 0 and 10. Gray lines indicate isotype control Representative data are shown out of 3 independent experiments using distinct donors (B) The percentages of BTLA positive cells were summarized in γδ T cells following the indicated stimulation. The first column shows the results of Day 0. Data are shown as mean±SEM of 3 independent experiments (Donor No. n=3). P = 0.0404 in γδ T cells before stimulation versus after 10 days’ stimulation of PBMCs with IL-2 and Zol; p = 0.0469 in γδ T cells before stimulation versus after 10 days’ stimulation of PBMC co-cultivated with HVEM^low^ Jurkat cells. (C) The percentages of PD-1 positive cells were summarized in γδ T cells following the indicated stimulation. The first column shows the results of Day 0. Data are shown as mean±SEM of 3 independent experiments (Donor No. n=3). P = 0.0417 in γδ T cells before stimulation versus after 10 days’ stimulation of PBMCs with IL-2 and Zol. Paired t-test was used. *p<0.05.

**Supporting Figure 6. Blockades of the BTLA/HVEM and PD-1 signaling do not affect the degranulation of γδ T cells.** In the presence of anti-human CD107a mAb, PBMCs were incubated overnight with WT or HVEM^low^ HL-60 cells. (A) The gating strategy to determine the proportion of CD107a^+^ γδ T cells. (B) Representative histograms showing CD107a expression in the γδ T cells. Gray lines indicate the isotype control. (C) The percentages of CD107a^+^ γδ T cells were assessed *ex vivo* with the designated cells and Ab in the absence of IL-2 and Zol. Data shown as mean±SEM of 1-2 independent experiments. (D) Cells were expanded in the indicated culture condition (See below the graph) for 14 days. In the presence of anti-human CD107a mAb, *in vitro* expanded γδ T cells were incubated overnight with the indicated target cells (Top). The percentages of CD107a^+^ γδ T cells were summarized as means±SEM of 2 independent experiments (Donor No. n-2).
