# Supplementary material for: The BTLA and PD‐1 signaling pathways independently regulate the proliferation and cytotoxicity of human peripheral blood γδ T cells
Source: Immun Inflamm Dis. 2020 Dec 17;9(1):274–87. doi: 10.1002/iid3.390 (PMC7860523; doi:10.1002/iid3.390)
Supplement: Supplementary file 2 — Supporting information. [file IID3-9-274-s002.pptx]

## Slide 1
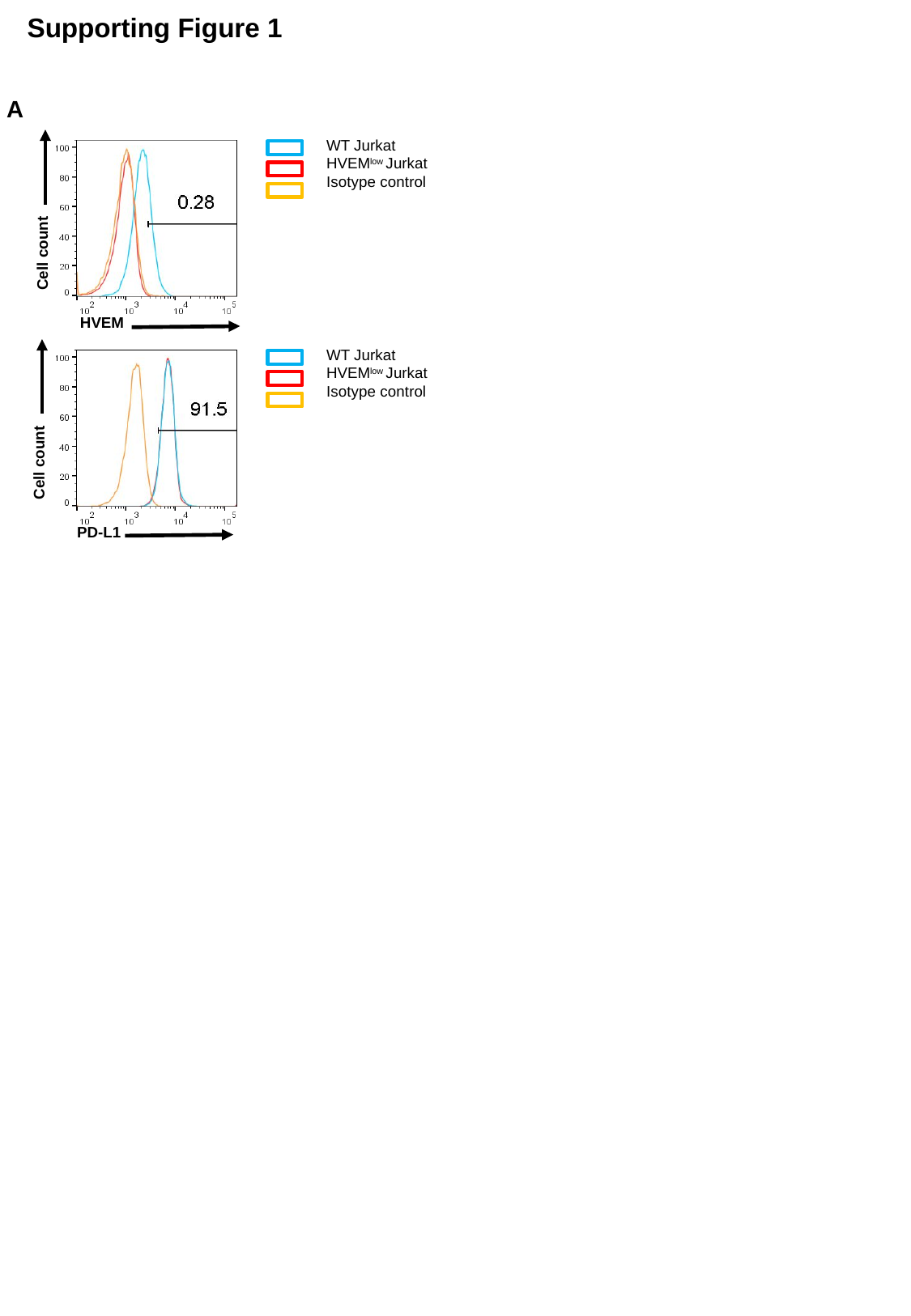

Supporting Figure 1
A
WT Jurkat
HVEMlow Jurkat
Isotype control
Cell count
HVEM
WT Jurkat
HVEMlow Jurkat
Isotype control
Cell count
PD-L1

## Slide 2
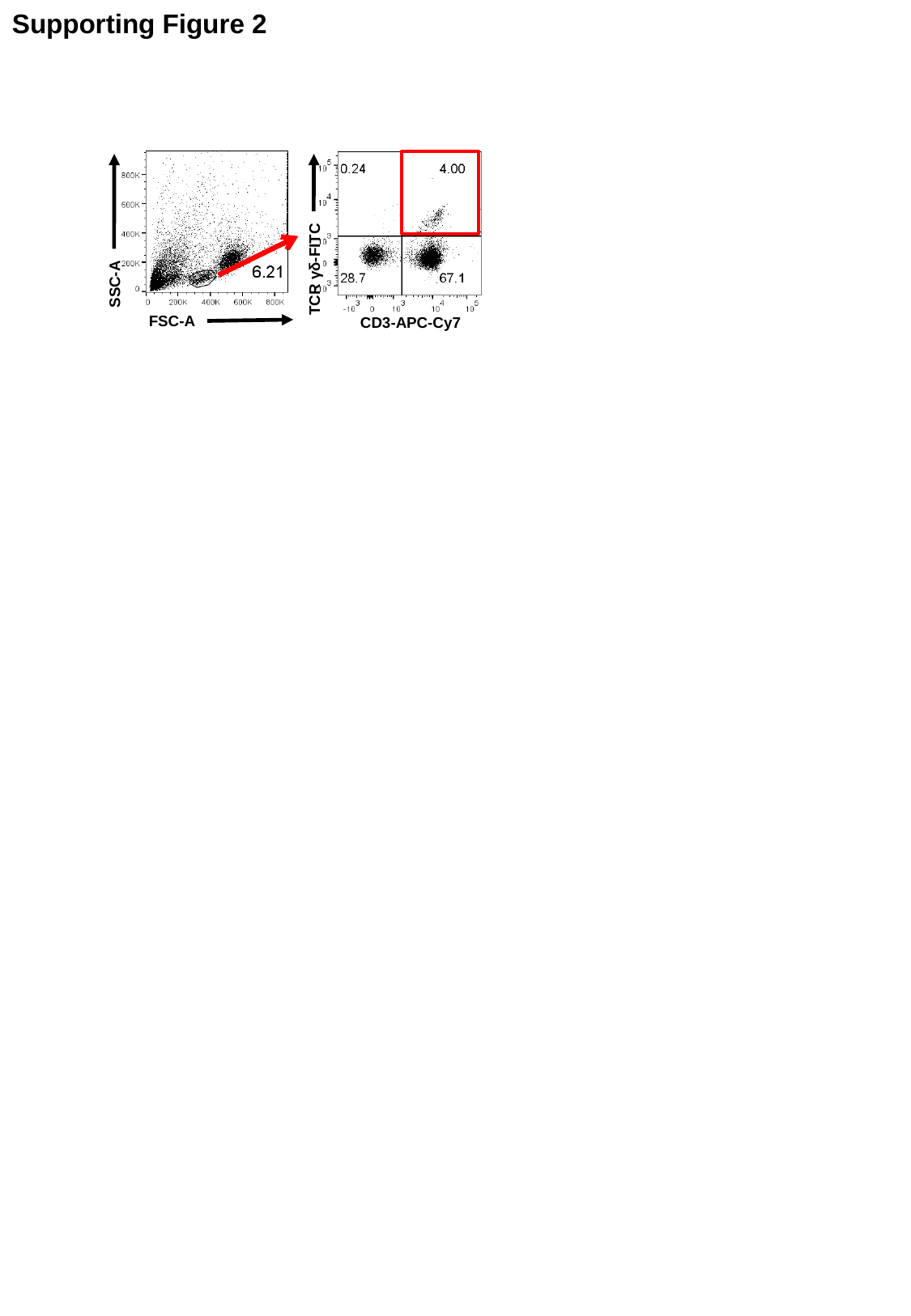

Supporting Figure 2
TCR γδ-FITC
SSC-A
FSC-A
CD3-APC-Cy7

## Slide 3
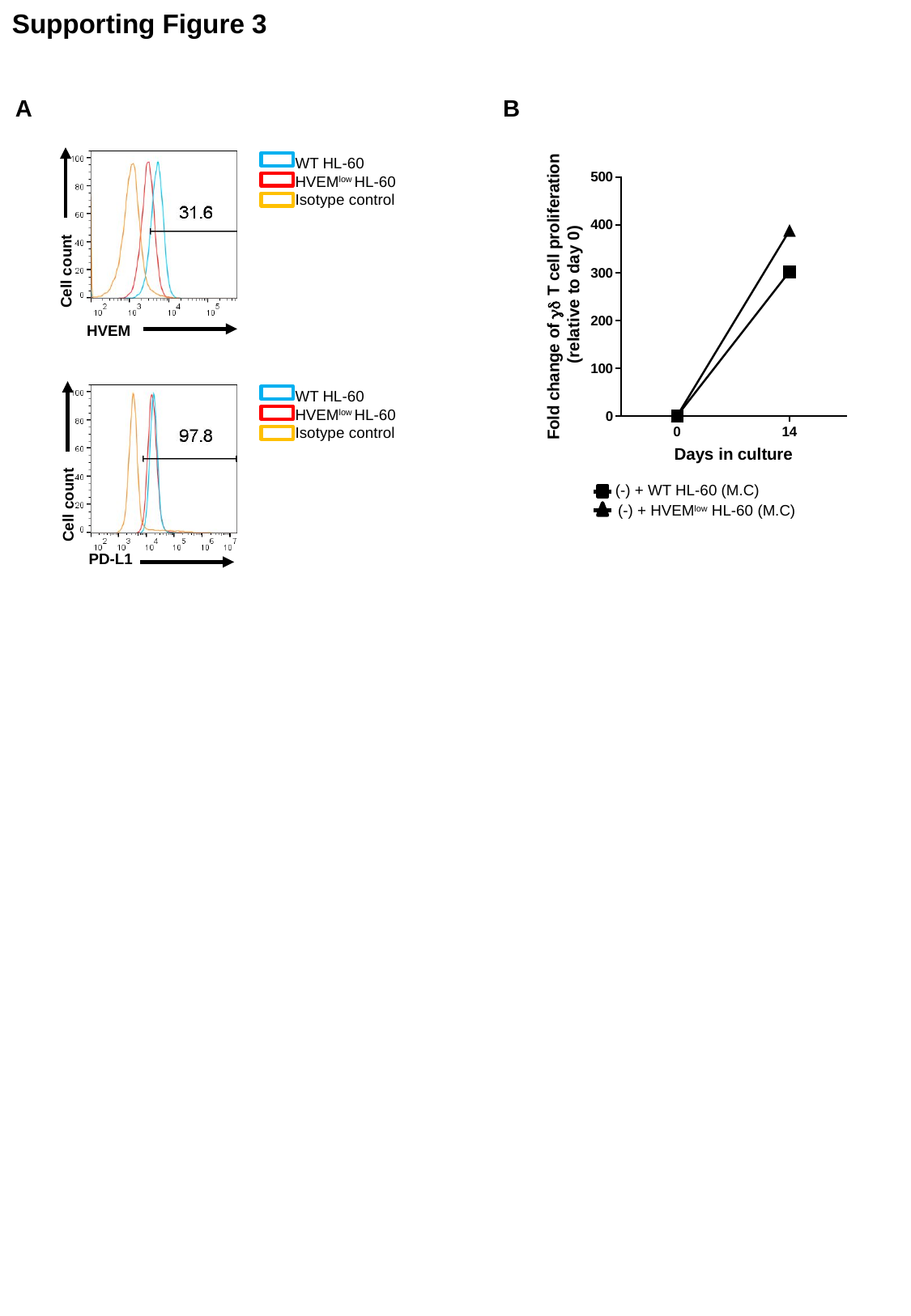

Supporting Figure 3
B
A
WT HL-60
HVEMlow HL-60
Isotype control
Cell count
HVEM
WT HL-60
HVEMlow HL-60
Isotype control
(-) + WT HL-60 (M.C)
Cell count
(-) + HVEMlow HL-60 (M.C)
PD-L1

## Slide 4
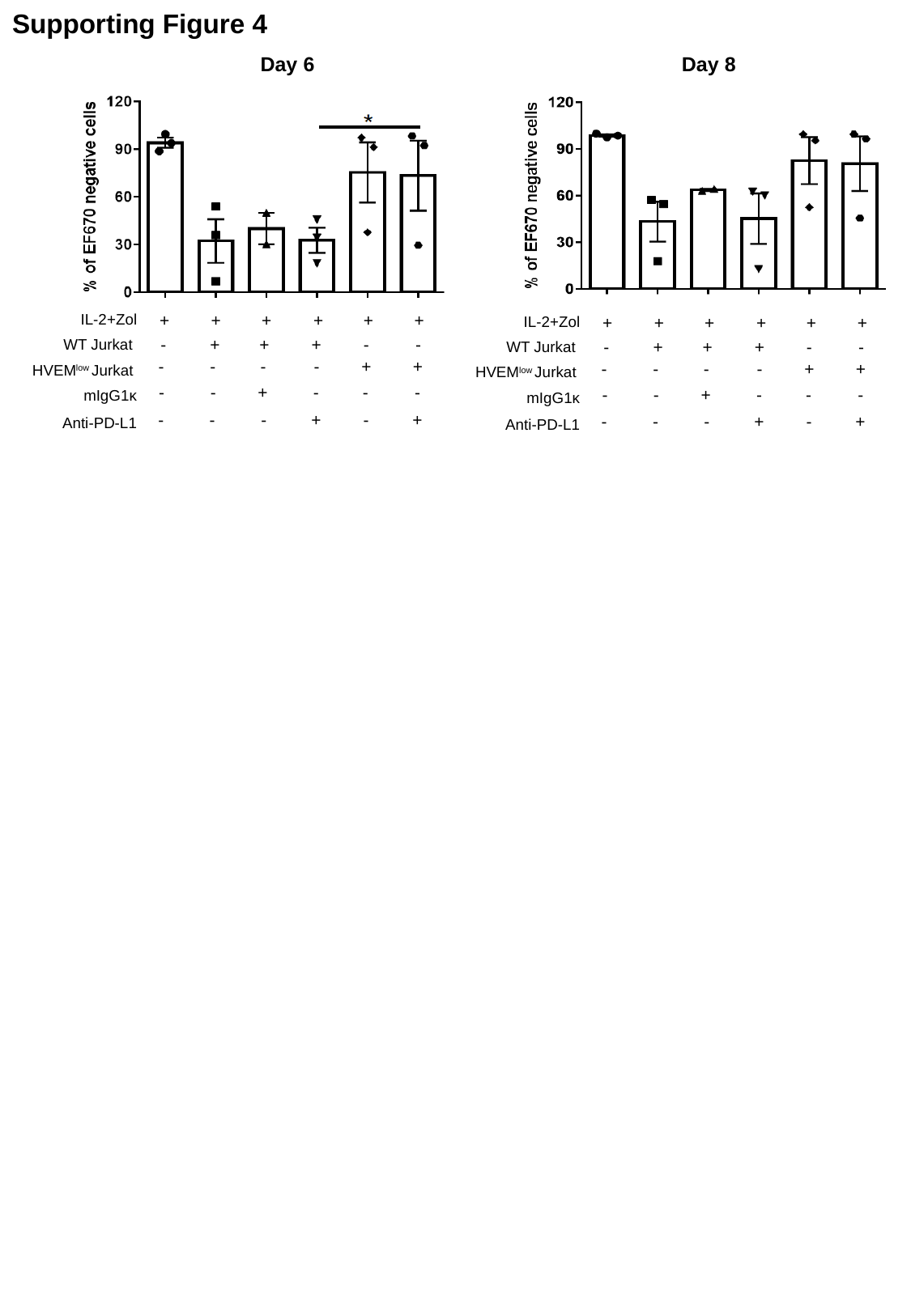

Supporting Figure 4
Day 6 Day 8
 + + + + + +
IL-2+Zol
 - + + + - -
WT Jurkat
- - - - + +
HVEMlow Jurkat
- - + - - -
mIgG1κ
- - - + - +
Anti-PD-L1
 + + + + + +
IL-2+Zol
 - + + + - -
WT Jurkat
- - - - + +
HVEMlow Jurkat
- - + - - -
mIgG1κ
- - - + - +
Anti-PD-L1

## Slide 5
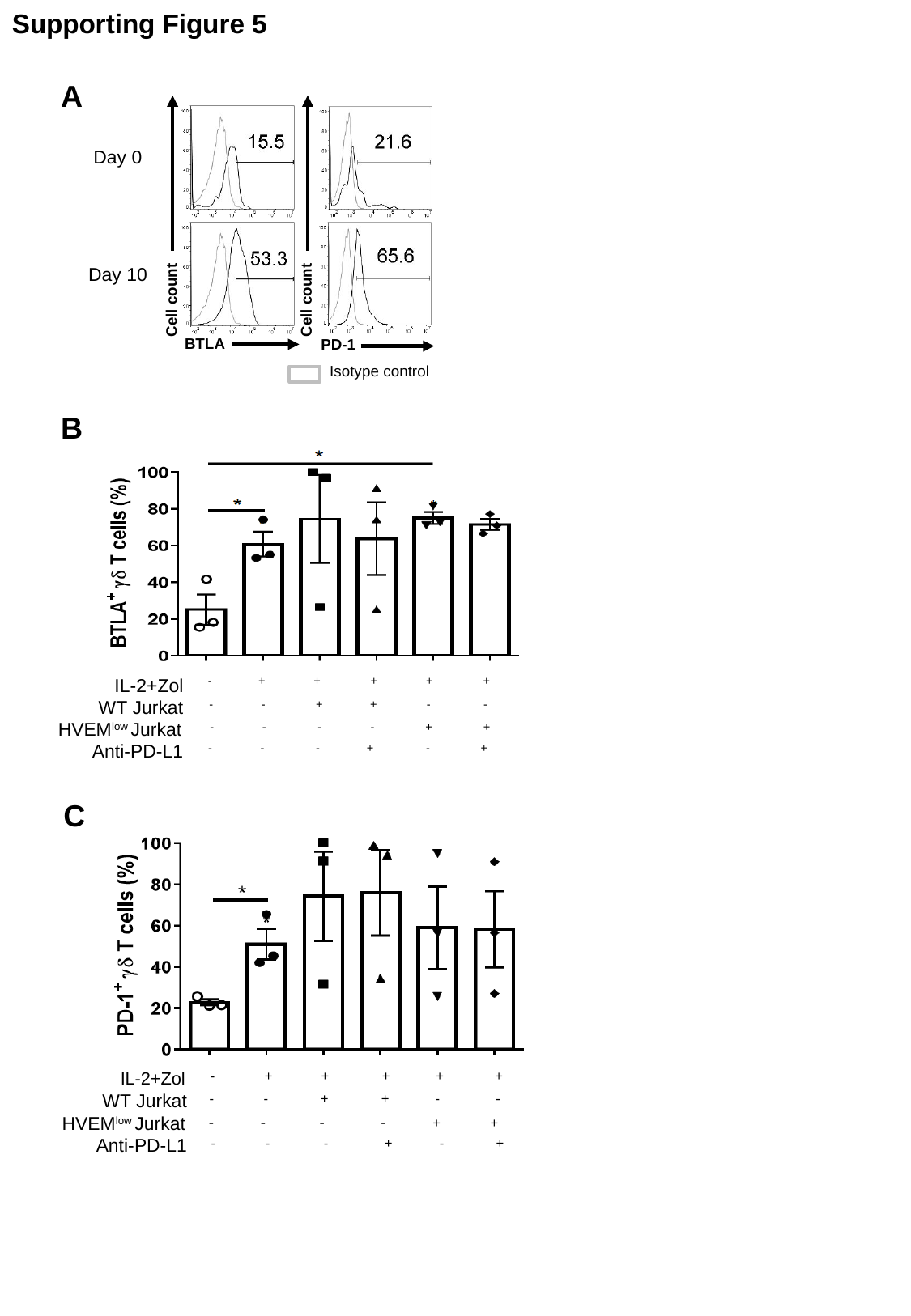

A
Supporting Figure 5
A
Day 0
Day 10
Cell count
Cell count
BTLA
PD-1
Isotype control
B
- + + + + +
IL-2+Zol
WT Jurkat
- - + + - -
HVEMlow Jurkat
- - - - + +
Anti-PD-L1
- - - + - +
C
- + + + + +
IL-2+Zol
WT Jurkat
- - + + - -
HVEMlow Jurkat
- - - - + +
Anti-PD-L1
- - - + - +

## Slide 6
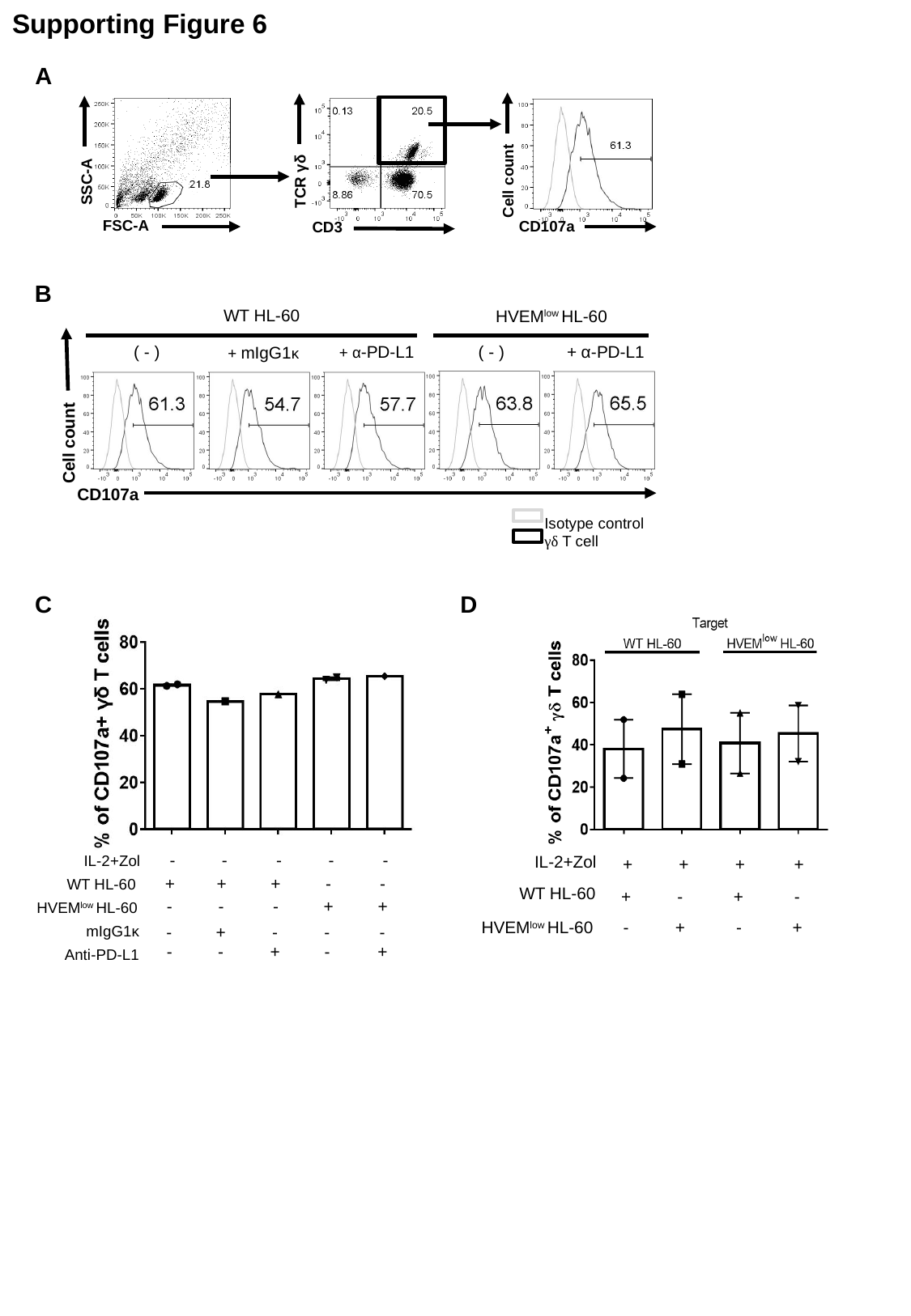

Supporting Figure 6
A
Cell count
TCR γδ
SSC-A
FSC-A
CD107a
CD3
HVEMlow HL-60
WT HL-60
B
+ α-PD-L1
+ α-PD-L1
+ mIgG1κ
( - )
( - )
Cell count
CD107a
Isotype control
γδ T cell
C
D
 - - - - -
IL-2+Zol
 + + + - -
WT HL-60
- - - + +
HVEMlow HL-60
- + - - -
mIgG1κ
- - + - +
Anti-PD-L1
IL-2+Zol
+ + + +
+ - + -
- + - +
WT HL-60
HVEMlow HL-60
